# College Nurses and the Bill

**Published:** 1919-02-22

**Authors:** 


*To the Editor of* The Hospital.

### College Nurses and the Bill.

*To the Editor of* The Hospital.

Sir,—Regarding the coming elections, for whom do we intend to vote? I should suggest, for whom should we like to vote? Personally, I should vote for the candidate who really means to exert herself in our behalf, and has the time and energy at her disposal for so doing. Some one practical; the person who realises that the day of fine speeches is over and the hour for practical reformation is at hand. Some one really representing us, one of us, the working nurse; this is my ideal. But has any one amongst us the time or the energy sufficient to devote to the looking after of our own affairs? We have neither. Are the governors of hospitals, nursing-homes, &c., either anxious or willing to provide us with the time sufficient to attend, perhaps travel to attend, meetings of the College or its branches? I doubt it, and consequently the working nurse is almost conspicuous by her absence on the executive of her own College.

Then, do the matrons and superintendents who monopolise a majority on the Council and on the Board, do they really represent the rank-and-file? Can they possibly see things from our standpoint? We can speak for the great majority, but are there any among them who by placing their own interests before the interests of those whom they are supposed to represent become a stumbling-block on the road to reform? We sincerely hope not, and we should be sorry even to think it. But are there? We cannot say—we do not know, we have no means of knowing; their work on our behalf to us is a closed book. He or she may or may not be all that can be desired, amply repaying the trust reposed in her, or she may be otherwise—we know not.

Now is the time for every member of the College to be up and doing. Select your candidates cautiously. Prove, as far as is possible to judge, their worth to our cause in the near future, when they will be called upon, and need all their reserve forces to fight, with our assistance, the enemies of the College, at present at large in the shape of a Central Committee's Bill. Carefully select a combination of brains, strength, and enthusiasm that, determined to let the world see we are in earnest, will make a bold front. Present this long-talked-of Bill of ours to the new Parliament, not merely to repose in the archives for a quarter of a century, nor yet to provide a companion for the Irish Home Rule Bill, but to go through. This accomplished, the coveted 20,000 members will be an easy matter. or 50,000 for the matter of that.

Should the Bill be rejected, and State Registration, according to its terms, be denied us, then we should be compelled, with or without the consent of the College, to take steps widely different from any ideals we have ever entertained, and, by taking matters in our own hands, bearing in mind the motto, "God helps those who help themselves," see what we can accomplish. But let us sincerely hope it will never be our lot to be compelled to have recourse to such measures, and should the day ever come when it will be necessary to take such a step, then the fault will not be ours, but lie at the door of those who deliberately and persistently refuse to "see."

Jan. 31, 1919. A Member of the College.

